# 

**Published:** 1874-09

**Authors:** 


### CONTENTS:

### ORIGINAL COMMUNICATIONS.

ARTICLE I.—Sixth Annual Session of the Southern Dental Association........ 193
ARTICLE II.—Record of Tests of Saliva. By Geo. H. Cushing........ 216
ARTICLE III.—Improved Method of Packing Moulds. By Dr. Geo. S. Fouke........ 222

### SELECTED ARTICLES.

ARTICLE IV Conservative Treatment of the Dental Pulps........ 224

### EDITORIAL, ETC.

The St. Louis Meeting........ 232

Useful Hints........ 233

Large Fees........ 234

### OBITUARY.

Dr. T. B. Hitchcock........ 234

### BIBLIOGRAPHICAL.

The Physiology of Man........ 235

### MONTHLY SUMMARY.

Experiments Upon the Human Brain........ 237
Preservation from Hydrophobia........ 237
Treatment of Poisoning by Chloral........ 238
Electricity for Chilblains........ 239
Cure for Toothache........ 239
Marine Glue........ 239
Ether and Chloroform........ 239
Singular Death from Blood-poisoning........ 239

